# Life in a Scotch Fever Hospital

**Published:** 1906-11-10

**Authors:** 


### LIFE IN A SCOTCH FEVER HOSPITAL.

### A PATIENT'S EXPERIENCE.

We print the following contribution because it shows how excellent from the patient's point of view are the arrangements at our great city isolation hospitals. It is to the interests of the whole community that this should be known and appreciated by every citizen.

My brother and I number among the poor unfortunates who have to live in lodgings. Shortly before Easter I was laid on my back by an attack of fever. My brother also fell ill at the same time. Our thoughts of Easter in the country were doomed to disappointment, for instead of getting better we got worse. The doctor soon came to the conclusion that we both had scarlet fever, and strongly advised us to go to one of the fever hospitals of our city. The ambulance van was to come on the Monday, but owing to some misunderstanding it did not turn up. Our landlady coming home about 10.30 P.M. and finding that we had not left, went off to the nearest telephone office and told them to come and fetch us as soon as possible. The van eventually arrived about 2.30 A.M. First to enter our bedroom was a porter carrying rugs and blankets, then a pretty hooded nurse. What a rare institution a nurse is; only those who have come in contact with them know to the fullest what a blessing they can be to those who are sick and suffering! We were cross-examined as to our age, occupations, home address, married or single (under the circumstances a rather unnecessary question), previous infectious diseases, and as to the history of each of our cases. It was thought best to put me on a stretcher. This was soon done, and, with the help of another porter, I was carried downstairs, my brother and nurse following.

We had an hour's drive, and arrived at Ward 19 just as the morning began to dawn.

The night nurse had everything ready, beds turned down, water-bottles in, and a hot drink of milk for each of us. There were twelve beds in our part of the ward, and from most of them came the sound of sleep. Our temperatures and pulses being taken, we were left to ourselves. Sleep was difficult; everything was new, and a strange subdued quietness seemed to pervade the ward.

Eventually I went off to sleep, but not for long. The boys began to wake up about half-past five, and soon the ward more resembled a boarding-school dormitory than the inside of a hospital. Most of the boys were convalescent and feeling perfectly fit.

Beds were made from six to seven. Breakfast then followed; the two day nurses came on at this hour. The majority of the boys had eggs, bread and butter, and tea. At 8.30 sister came on duty, and two hours later our ward doctor made his appearance. He sounded us both and moved on. Most of the boys said they were "fine" in answer to the doctor. I wished that I could have said the same thing. At twelve we had dinner; there was always soup, chicken, and some kind of milk pudding. Tea at four, supper at six, when every boy had a bowl of porridge.

In the evening another boy arrived, a little chap about ten, who cried for about twelve hours for his mother. He at length came to the conclusion that it was no good "greeting," as the boys called it, and settled down to make the best of it. It may seem strange, but this boy had come from one of the poorest quarters, and the night before his little sister had been slept upon and killed, and yet he cried to go home. His arrival brought our ward up to the limit. There were eighteen; most of them were boys from eight to fourteen, there were two working men, a hospital porter, a young apprentice engineer, and a Norwegian, who was on his way to America. He could speak a little English and was very pleasant company.

By the first evening it seemed ages since the morning. The first day was the longest, and we soon got accustomed to the life in hospital. In the evenings we usually played whist; during the day one chatted and played games—chess, draughts and halma. Post arrived about eleven, and was always looked forward to; it was one of the events of the day. Life in a hospital, to those who can suit themselves to their surroundings, is anything but an unhappy one. There is plenty of fun, and the days pass by very quickly. After being in bed four weeks—this is the time which every patient must remain in bed—my brother was allowed up. I was, however, suffering from rheumatism so badly as to remain in bed. Several times during our first few weeks a number of medical students, both male and female, came round the wards. They seemed to bring a touch of the outside world with them. Judging from their faces, they all seemed to enjoy their afternoon's experience in the fever wards. At the end of nine weeks I was permitted to get up, and a few days after went into the grounds. It is only when one gets outside that you can fully realise how isolated you are. You walk along one road and come to a notice: "No scarlet fever patients to pass this gate," and the same along another. On three sides is an unclimbable fence. In the distance you see people going into the city, children playing, and everybody going and coming as they please; it is only when you view all this that you feel like a prisoner and have a feeling to rebel against it, and yet know how feeble you are.

In a fever hospital it is usual for the patients to help the nurses. They clean the brass fittings, polish the floors, wash down the tiles, bring in the plants, and other similar work. It may not be usual, but in the hospital we were in Sunday is like any other day, the only difference being that the nurses have longer time off duty, and one can hear bells ringing. There was no service in the wards. After being an inmate for nearly thirteen weeks I was passed by the doctor, and the following Saturday I said "Good-bye." It may be of interest to hear of the way one changes from a fever patient to an ordinary individual.

The baths are stationed near to the exit. On the one side you are in the prohibited part of the grounds, but on the other you can go where you please. The bath-rooms, to the number of about twenty, are arranged along a corridor, and each one is divided into three parts. Firstly you enter a small dressing-box. Here you take off your hospital garments and go into the next compartment, where you find the bath ready for you. The water contains disinfectant, and one does not wish to stay in long. Having dried, you go into the third division; here you find your own clothes. It is a strange feeling to wear a linen collar and to assume once more your ordinary clothing. You leave your cubicle for a dressing-room, where you vainly try to make your hair lie down, for it will persist in standing on end, like the back of a dog when angered. The noise of the streets and the rush that everyone seems to be in are very noticeable, and somewhat trying at first. You feel as if everyone knew where you had come from, and you almost expect them to point their fingers at you. From my experience I should advise all people, no matter what their station in life, if ever taken with scarlet fever not to hesitate, but to go to an isolation hospital. The experience when all is over is a happy one, and it enables you to see a side of life which everyone does not know and which few understand.

